# Overcoming Addictions, a Web-Based Application, and SMART Recovery, an Online and In-Person Mutual Help Group for Problem Drinkers, Part 2: Six-Month Outcomes of a Randomized Controlled Trial and Qualitative Feedback From Participants

**DOI:** 10.2196/jmir.5508

**Published:** 2016-10-04

**Authors:** William Campbell, Reid K Hester, Kathryn L Lenberg, Harold D Delaney

**Affiliations:** ^1^ Behavior Therapy Associates, LLC Research Division Albuquerque, NM United States; ^2^ Truman Health Services University of New Mexico Albuquerque, NM United States; ^3^ Psychology Department University of New Mexico Albuquerque, NM United States

**Keywords:** alcohol addiction, intervention study, psychological techniques, digital interventions, engagement, online program, self-help

## Abstract

**Background:**

Despite empirical evidence supporting the use of Web-based interventions for problem drinking, much remains unknown about factors that influence their effectiveness.

**Objective:**

We evaluated the performance of 2 resources for people who want to achieve and maintain abstinence: SMART Recovery (SR) and Overcoming Addictions (OA). OA is a Web application based on SR. We also examined participant and intervention-related factors hypothesized to impact clinical outcomes of Web-based interventions.

**Methods:**

We recruited 189 heavy drinkers through SR’s website and in-person meetings throughout the United States. We began by randomly assigning participants to (1) SR meetings alone, (2) OA alone, and (3) OA and SR (OA+SR). Recruitment challenges compelled us to assign participants only to SR (n=86) or OA+SR (n=102). The experimental hypotheses were as follows: (1) Both groups will reduce their drinking and alcohol-related consequences at follow-up compared with their baseline levels, and (2) The OA+SR condition will reduce their drinking and alcohol or drug-related consequences more than the SR only condition. Additionally, we derived 3 groups empirically (SR, OA, and OA+SR) based on the participants’ actual use of each intervention and conducted analyses by comparing them. Primary outcome measures included percent days abstinent (PDA), mean drinks per drinking day (DDD), and alcohol or drug-related consequences. Postbaseline assessments were conducted by phone at 3 and 6 months. Secondary analyses explored whether clinical issues (eg, severity of alcohol problems, level of distress, readiness to change) or intervention-related factors (eg, Internet fluency, satisfaction with site) affected outcomes.

**Results:**

Both intent-to-treat analyses and the actual-use analyses showed highly significant improvement from baseline to follow-ups for all 3 groups. Mean within-subject effect sizes were large (*d*>0.8) overall. There was no significant difference between groups in the amount of improvement from baseline to the average of the follow-ups. We found that participants who stopped drinking before joining the clinical trial had significantly better outcomes than participants who were still drinking when they joined the study. Neither Internet fluency nor participants’ reported ease of navigating the site had an impact on outcomes.

**Conclusions:**

These results support our first experimental hypothesis but not the second. On average, participants improved on all dependent measures. Both SR and OA helped participants recover from their problem drinking. Web-based interventions can help even those individuals with lengthy histories of heavy drinking to make clinically significant reductions in their consumption and related problems. These interventions work well for individuals in the action stage of change.

**Trial Registration:**

Clinicaltrials.gov NCT01389297; https://clinicaltrials.gov/ct2/show/NCT01389297 (Archived by WebCite at http://www.webcitation.org/6kLNUNDcc)

## Introduction

### Background

Clinicians, researchers, and public health officials working to reduce the prevalence of substance use disorders have sought to develop and implement a range of evidence-based treatments and techniques (EBTs) over the last 20 years. Concomitant with these clinical developments, the emergence and growth of personal computing, media technology, and the Internet afforded new means and contexts for the adaptation and delivery of EBTs. In fact, apps and Web-based interventions for hazardous drinking have proliferated over the last decade [1-3].

With regard to their ability to deliver clinical services, Web-based interventions in general have several proven as well as potential virtues. Accuracy and validity of assessment protocols, probabilistic feedback algorithms, reliable computations, impartial results and objectives, and tailored recommendations are all appropriate functions of computers, making them (theoretically) optimal for the delivery of evidence-based behavioral health interventions [4-9]. The ubiquity of the Internet and mobile technology afford these interventions with a greater accessibility and reach, on both an individual and public level, and theoretically their impact on public health could be significant. However, the nature of the medium and the way people use Web-based interventions present serious challenges to the field. While developers have control over the content and design of the program, the remote context of implementation affords users a great deal of freedom in how they actually engage with the intervention and also precludes close assessment of ostensible therapeutic mechanisms [10,11]. Further, people often exhibit significantly less engagement with Web-based interventions than developers envision when they design them [11-13]. Indeed, there is a substantial proportion of users who drop out of programs after a single visit to a site [14,15].

When subjected to empirical tests of their effectiveness, self-guided Web-based interventions for problematic alcohol and substance use have consistently exhibited effect sizes that range from medium to disappointingly low [1,4,16-19]. Questions persist as to whether there is in fact a dose-response effect of engagement (ie, greater use of a site is associated with better outcomes), and if so, what can be done to enhance engagement with any given Web-based intervention to increase its effectiveness. The evidence on the relationship between engagement and outcomes is equivocal, with some reviewers and researchers finding support for a connection [20-22] and others finding no such evidence [15,23-25]. Nonetheless, much effort has gone into determining what factors (whether related to the users of the program or the programs themselves) influence adherence and engagement with Web-based interventions [2,14,25]. The general consensus among clinicians and researchers in the field is that until and unless the puzzle of engagement is solved, the seeming potential of Web-based interventions will not be fully realized.

Thus, as studies have accumulated over the last 10 years, interest has grown in identifying factors that might influence engagement with, and the effectiveness of, these interventions [2,3,14,26]. Leading investigators agree that more needs to be known about how EBTs are best adapted to Web-based format (intervention-related factors) as well as who is most likely to use and benefit from access to such a format (participant-related factors) [2,10,12,14,21]. There have also been calls for researchers to cleave to a rigorous set of standards in the development and testing of Web-based interventions (ie, to clearly report the study’s rationale, methods, and limitations) and do what they can during clinical trials to explore both intervention-related and participant-related factors that are thought to influence outcomes [1,2,10,14,27,28].

### The Study

In this randomized controlled trial, we evaluated the performance of 2 resources for heavy drinkers: SMART Recovery (SR) and Overcoming Addictions (OA). OA is an online intervention that we developed based on the principles and practices of SR. There were 2 main goals of this study. First, we sought to determine whether SR and OA helped individuals make clinically significant reductions in their alcohol consumption and related problems. In addition, we were also interested to know if participants with access to OA would experience better outcomes than those assigned to SR. Second, we wanted to know more about who was most likely to engage with and benefit from these online resources and whether there were factors related to the site that influenced engagement and outcomes. Overall, we sought to learn more about translating EBTs into Web-based programs, and in the process to develop a more effective empirically supported intervention for drug and alcohol misuse.

We chose SR [29] as the model for our intervention because of its sound theoretical orientation, its commitment to EBTs, and, pragmatically, because the cognitive-behavioral exercises found in SR are well suited to online dissemination. SR’s program uses a common set of cognitive and behavioral strategies to address all addictive behaviors [30]. Their rationale for this is based on the generally accepted theory that common etiological factors underlie the development and maintenance of addictive behaviors (eg, stimulus control, maladaptive reinforcers) as well as the broad applicability of cognitive-behavioral and motivational strategies that are supported by outcome research in the treatment of various addictions. The outcomes of individuals who visit the SR website have never been subjected to empirical analysis before this study, but because SR is explicitly based on the use of cognitive-behavioral EBTs, we hypothesized that people who visited the site would, on average, change their drinking.

OA is designed to be used either as a complement to traditional SR (ie, meetings and workbook) or as a stand-alone, self-guided program. We thought that a structured, self-guided site, providing an enhanced suite of SR exercises and entailing the benefits of Web-based interventions (ie, accessibility, reliability, interactivity), would improve outcomes for individuals seeking SR. Although at the time of this study SR did offer a workbook, OA’s comprehensive structure brought SR’s exercises and rationale into an organized, integrated program. We assumed that individuals would benefit from being able to access program content at any time and from any place that was most convenient to them, rather than, for example, having to navigate the scheduling constraints of SR meetings. We reasoned that OA’s consistent and clinically valid presentation of treatment components (ie, the concepts being taught or the exercises offered) would increase their effectiveness. We also thought that unique features of the site such as the ability to track and get feedback on triggers and urges to drink, guided mindfulness exercises, and the videos provided by highly regarded SR trainers, would enhance engagement with therapeutic mechanisms and likewise lead to better outcomes.

At the same time, we were interested in learning more about who was most likely to benefit from OA and whether the design of the site was impacting outcomes. The issue of matching clients to interventions is as important to Web-based interventions as it is in the context of face-to-face treatment. Research has found that factors related to characteristics such as gender, age, level of education or income, level of alcohol consumption, and readiness to change all contribute to Web-based treatment adherence [31,32], although the influence is complex [15] and evidence is as yet inconclusive regarding their influence on outcomes on various clinical measures [1,33].

Another individual factor that has received some attention in trials of Web-based interventions is “readiness to change.” The Transtheoretical Model of Change [34] has long been recognized for its ability to inform behavioral treatments [35,36] and has in fact been used in the development of Web-based interventions as a theoretical basis for the tailoring they provide [5,37]. Like any self-directed program, Web-based interventions are ostensibly well suited for individuals in the action stage of change [11]. Prior research has shown that Web-based interventions can increase readiness to change [37], and one study found that individuals who were high in treatment readiness (ie, approaching the “action” stage of change) were more likely to complete a Web-based program [28]. It has yet to be shown empirically that such individuals do in fact benefit from Web-based interventions and, conversely, whether individuals who are still in the contemplation stage of change can also benefit from them. Evidence to support establishing such a distinction for individuals seeking to change their drinking could inform treatment recommendations as well as implementation strategies for Web-based interventions more broadly.

A person’s relative ability to function effectively with computers and on the Internet is another individual factor of interest to Web-based intervention developers. Feasibility studies have consistently found that site visitors are quite conscious of the difficulty they experience navigating Web-based programs [32,38-41] and may disengage from the programs if the process of using them becomes too frustrating [41-43]. As one way of assessing this difficulty, researchers have examined whether users’ relative proficiency with “Internet skills” can moderate their ability to benefit from a Web-based intervention [38-40]. While we know people can struggle to effectively navigate websites and so might fail to obtain relevant information in ways that interfere with Web-based interventions, there is as yet no evidence to support the theory that such difficulties moderate clinical outcomes. Nonetheless, it seemed reasonable to assume that participants who typically spent more time on the Internet and who navigated the site with more ease would have more facility with the program, and so derive greater benefit from the treatment it conveys.

Considering the participant factor of Internet proficiency, we were curious whether there were aspects of OA’s design that would influence outcomes in ways not associated with the traditional delivery of EBTs (ie, face-to-face). Specifically, we were interested in participants’ subjective sense of how difficult the OA site was for them to navigate (ie, to successfully access the information and exercises in the program). With regard to navigating through course content, evidence shows that low prior-knowledge and low metacognitive learners learn better when the program dictates the pacing and structure of the content (ie, utilizing guided information architecture) rather than leaving it to the learner to decide how to proceed [44]. According to e-learning researchers, novice learners don’t know enough about a given domain to benefit from “learner control” over the structure and pacing of the content [45].

Furthermore, one common assumption about Web-based interventions is that persuasive features such as site architecture and navigation, the use of video or social media, or the deployment of email or text messaging prompts can positively impact engagement with therapeutic mechanisms [13,26,32,43]. On the other hand, researchers have found that providing too much content can depress engagement with Web-based programs [46]. The principle applies whether content is added to enhance interest [47], to increase depth [48,49], or to expand on key ideas [50,51]. Given the cognitive impairments commonly associated with early recovery from hazardous drinking, we sought to know whether the user’s subjectively rated satisfaction with their ability to navigate the website, as well as the amount of content on the site, would account for variance in outcomes.

Finally, with regard to the optimal methods for conducting a clinical trial on line, among those who develop and validate Web-based interventions, there is a well-known trade-off between more ecologically valid and more clinically rich methodologies [10]. There have been several clinical trials conducted entirely online, the method generally regarded as the most ecologically valid, and so most indicative of “real-world” effectiveness [10,11]. However, in addition to the disadvantage of their typically high rates of attrition, such tests are constrained in their ability to gather data regarding factors that might influence outcomes [10,13,14]. Indeed, it is often impossible to know who the participants are, whether their reports are genuine, and how seriously they are treating the intervention.

For our study, we chose to recruit from individuals who were actively seeking information online about SR. We did this intentionally to test SR and OA with a sample drawn from the population that would likely be interested in SR’s treatment approach or EBTs more generally. Furthermore, we chose to conduct our intake and follow-up interviews online and over the phone. Although even this limited contact with our research staff represents a significant deviation from the conditions under which self-guided Web-based interventions are encountered at large, online, we considered it a necessary trade-off in order to gather the data we needed for our qualitative analysis. We sought to minimize the impact of our contact with participants on our findings by conducting our baseline assessments at the three-month follow-up.

## Methods

### Trial Design

Further details regarding study recruitment, inclusion and exclusion criteria, the screening process, randomization, assessments, baseline and follow-up interviews, and institutional review board approval are presented in Part 1 of this study [23].

### Treatment Conditions

#### SMART Recovery

SR is a nonprofit educational organization run by a board of directors that consists primarily of clinicians with backgrounds in cognitive-behavioral practice [28]. The board determines, based on the empirical evidence and the feasibility of translating elements into self-help formats, what components will make up the SR program. While SR content may vary as empirical research evolves, the underlying philosophy of the protocol has remained consistent since its inception. In particular, SR promotes the dissemination of, and instruction in, empirically supported techniques and practices that “empower” [28] individuals to make changes in their own lives.

SMART Recovery’s program for change is focused on the following 4 domains: (1) building and maintaining motivation for change; (2) dealing with urges; (3) managing thoughts, feelings and behaviors; and (4) developing a balanced lifestyle. To build motivation, SR offers such exercises as cost-benefit analysis and guidance on how to develop a change plan. With regard to dealing with urges, SR teaches individuals how to identify and think functionally about triggers as well as how to manage urges when they arise. SR prescribes the use of such cognitive interventions as disputing irrational beliefs, and the “ABC” exercise commonly used to understand and improve upon emotional upsets. SR also provides instructions for learning relapse prevention techniques. Finally, to help support lifestyle changes that coincide with changes in drinking, SR offers exercises designed to help individuals identify and plan for meaningful activities, attain a balanced life, and engage in healthier behaviors.

SMART Recovery has been implemented historically in face-to-face and online self-help or mutual aid groups, with meetings that are facilitated only by individuals who have received official SR training [28]. While there is no formal treatment manual for SR, interested individuals obtain a workbook containing various descriptions of SR principles and exercises. The SR website serves as a resource for individuals who are seeking information about, or are actively engaged in, addressing their alcohol or drug use through SR. The site explains the principles of SR in detail, contains resources to support SR exercises, and serves as a portal for an SR community, including contacts for in-person meetings around the country, live online SR meetings, and a blog. While the site contains a wealth of resources pertaining to SR, it does not provide a Web-based SR intervention, nor does it expressly advise site visitors about how to utilize SR’s treatment components.

#### Overcoming Addictions

OA is a self-directed Web-based intervention designed for individuals who want to stop drinking and are in the “action” stage of change [34]. It is intended to faithfully render the EBTs of SR while also enhancing engagement with its therapeutic mechanisms. Once visitors register to use the site, the program creates a new record in the database, personalizes the content, and directs them to the homepage. The content is organized into “modules” around the 4 points of the SR program. The site also contains exercises not found in the SR handbook or website (eg, mindfulness and meditation exercises), but which are empirically supported and that we judged to be consistent with SR’s 4-point program.

For example, the first module, *Getting Started*, provides an overview of the program and its theoretical approach, while also explaining Stages of Change, and addressing how an individual’s relative stage might influence their approach to the program. The next module, *Building and Maintaining Motivation for Change*, begins with an exercise to help individuals think about how their drinking and their desire to change relates to their values; it then proceeds to a decisional balance exercise that helps users to consider the pros and cons of changing. The third module, *Dealing with Urges and Cravings*, contains information about urges and triggers and provides users the tools to monitor, track, and develop strategies to handle them. The fourth module, *Self-Managing Thoughts, Behaviors, and Feelings* contains common cognitive behavioral therapy exercises such as problem solving, functional analysis of problematic behaviors and situations, and information about the interactions between thoughts and feelings that may influence drinking. The final module, *Lifestyle Balance for Preventing Relapse*, has exercises that support regaining one’s health, learning relaxation techniques, goal setting, and relapse prevention strategies. In order to support self-guided use, we included videos recorded by experienced SR facilitators explaining how to think about and use the various exercises presented on the site. We also included a graphic feedback features wherever appropriate (eg, feedback on changes in urges to drink over time) and the ability for users to save their work and track their progress through the site.

Structurally, we sought to create a site that emulates the philosophy of the intervention. In order to reflect SR’s emphasis on autonomy and self-direction, we originally designed the site in an open, unguided format so that a user could access any section or module of the program in any order that he or she believed would best suit the needs of their treatment. However, the site also reflects findings that guided “breadcrumb” navigation works best on behavior-change websites [32,42,52]; thus, once the user chooses an exercise, the program guides them through it in “tunnel” fashion, with a link on every page that leads to the next step of the exercise. We felt this “hybrid style” (ie, matrix to tunnel, and back to matrix) offered the best compromise between an unguided “user-centered” approach and the more directive protocols often used in conventional cognitive-behavioral interventions.

We made additional content available throughout the site via pop-up boxes and links that expand the page (eg, read more>>) to reduce the amount of visible content. We wrote the content for an 8^th^ grade reading level and confirmed that level with the Flesch-Kincaid readability test [53] (built into the Corel *WordPerfect* program). Images only appeared in the page headers that also included navigation links to the home page, module headings, and the My Account page where the text and email features are located.

### Group Allocation

In the initial design of the trial, we intended to randomly assign participants who were new to SR to one of the 3 conditions: (1) to use the online resources of SR and their meetings (face-to-face and/or online); (2) to the SR resources plus access to the OA Web app; or (3) to use the OA Web application only. However, as reported in Part 1 [23], we discovered that many potential study participants were disinclined to enroll in the study when they learned that they might be randomized to the OA only group. Because the majority of individuals came to the study after seeing the announcement on the SR website or by attending an SR meeting, they were unwilling to risk giving up the option to attend meetings for the sake of joining the study. After months of confronting this challenge, we ceased randomizing participants to the OA only group and decided to conduct separate posthoc analyses on the original 3 groups, derived empirically based on their actual use of SR and OA. We felt that even this nonrandomized analysis of participants’ treatment of choice would render useful data. We dubbed our modified original analyses “intent-to-treat” and our posthoc analyses “actual use.”

### Primary Analysis

Primary analyses of between-group differences were conducted to detect the effect of OA. Consistent with intent-to-treat analyses, we examined changes within the randomly assigned groups, using repeated measures analysis of variance as well as mixed model analyses, which were used for both tests of null hypotheses and tests of non-inferiority. The primary dependent variables were percent days abstinent (PDA), mean standard drinks per drinking day (DDD), and alcohol-related problems measured by the Inventory of Drug Use Consequences (InDUC) [54]. We used one three-level repeated factor (time of assessment: baseline, 3-month, 6-month), and the between-subject factor of treatment condition. For each analysis, 2 contrasts in the within-subject factor of time were conducted.

Our secondary analysis explored whether participant characteristics, including readiness to change and Internet skills, were associated with outcomes. Further, we tracked the extent to which participants used SR and OA, asked them to rate OA’s structure and complexity, and examined whether these indicators of engagement with the intervention were associated with outcomes.

### Secondary Analysis

The data for the analysis of the participant and intervention factors thought to impact the use of the intervention were collected in a semistructured exit interview at the six-month follow-up. We asked participants to estimate how much time they spent on the Internet each week. We also asked them to report on any steps they had taken to change their drinking over the course of the clinical trial and to attribute the relative benefit of any factors that helped them. In the OA group, participants were asked to rate the website with Likert scales across several dimensions, including how easy or hard the site was for them to navigate, whether the site’s structure helped or hindered accessing its treatment content, and whether they were satisfied with the amount of content on the site.

To test for the impact of these factors, separate repeated-measures analyses of variance were conducted on the 3 primary dependent variables (ie, InDuC, PDA, and DDD), with 2 within-subject continuous variables (eg, hours per week spent on the Internet; ease of use) and one within-subject dichotomous variable (amount of information: right or wrong) entered as covariates, and one three-level repeated factor (time of assessment: baseline, 3 month, 6 month). Again, for each analysis, we conducted 2 contrasts of the within-subject factor of time.

## Results

### Sample

**Figure 1:**
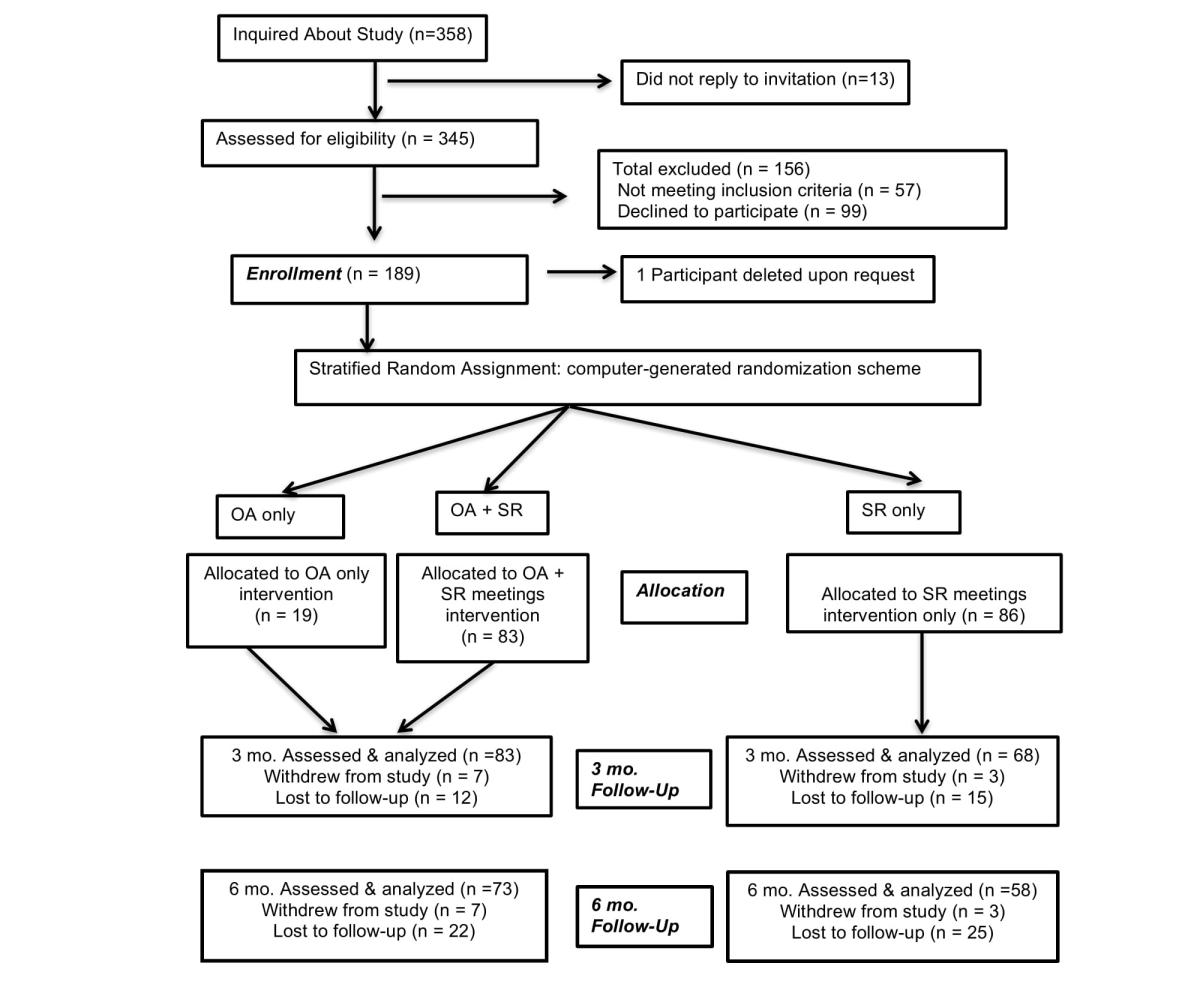
Consort study participant flow chart. OA: Overcoming Addictions; SR: SMART Recovery.

Figure 1 shows the CONSORT flow of participants through the study. A total of 358 people new to SR inquired about the study and of those 345 agreed to be screened. During the initial screening, 19 failed to meet the inclusion criteria and 38 were excluded. After passing the screen, 99 potential participants did not complete the initial consent process, 6 more failed to follow through with the initial assessment, and one asked to be dropped from the study within a day of being randomized. This resulted in 189 individuals who were randomly assigned to one of the 3 groups: SR, OA, and OA+SR. As noted above, due to complications of recruiting through SR’s network, the final allocation tallied 102 participants in the OA+SR and OA only groups and 87 in the SR group. Recruitment began from September 12, 2011 (3 pilot participants were recruited in the first 2 weeks of the study) and ended on August 1, 2012. Three-month follow-ups were completed on November 1, 2012. Six-month follow-ups were completed on March 14, 2013.

### Participant Characteristics

The general characteristics of the study participants as a whole and by group assignment are presented in Table 1. On average, the sample endorsed clinical levels of psychological and alcohol-related problems. The mean score on the Brief Symptom Inventory (BSI) of 17.4 (SD 12.9) indicates that a majority of participants were experiencing significant depressive, anxious and/or somatic distress at screening. Mean scores on the Alcohol Use Disorders Identification Test (AUDIT)=24.7 (SD 8.1), InDuC Lifetime=31.0 (SD 7.2), and InDuc Recent=41.4 (SD 17.9) indicate that many individuals were at the more severe end of the use disorder spectrum. The majority of the sample was female (61%), which is almost twice the prevalence rate for women in the United States [55], although this level of participation by women is common in eHeath clinical trials for alcohol problems [1,33]. There were no significant differences between groups on any variable. Finally, the sample is remarkably homogeneous with regard to race (90% white).

**Table 1:** Participant characteristics.

Group	Whole sample	OA^a^+SR^b^	SR only
Participants, n (%)	188	102 (54%)	86 (46%)
Female, n (%)	114 (60.6%)	62 (61%)	52 (61%)
Age in years, mean (SD)	44.3 (10.9)	45.3 (10.7)	43 (10.6)
White, n (%)	170 (90.4%)	94 (92.2%)	76 (88.4%)
Education in years, mean (SD)	16.1 (2.4)	17.7 (2.2)	15.9 (2.5)
BSI^c^ total, mean (SD)	17.4 (12.9)	15.7 (13.1)	19.4 (12.5)
AUDIT^d^, mean (SD)	24.7 (8.1)	24.6 (8.1)	24.8 (8.1)
InDuC^e^ Lifetime, mean (SD)	31.0 (7.2)	30.8 (6.7)	31.3 (7.7)
InDuC Recent, mean (SD)	41.4 (17.9)	40.6 (17.1)	42.2 (19.1)

^a^OA: Overcoming Addictions.

^b^SR: SMART Recovery.

^c^BSI: Brief Symptom Inventory.

^d^AUDIT: Alcohol Use Disorders Identification Test.

^e^InDuC: Inventory of Drug Use Consequences.

### Lost to Follow-Up

We compared baseline characteristics between those having complete data and those missing either or both of the 3-month and 6-month follow-ups. No significant differences were found on the following continuous variables at baseline: age, the AUDIT, BSI, InDUC scores, DDD, or PDA. No differences across groups existed on the categorical variables of group assignment, gender, or race. Only education level demonstrated a significant difference—those who provided data at both follow-ups reported having completed more years of education (16.4) than those who did not (15.5), *t*_186_= 2.24, *P*=.03.

### Intent-to-Treat Analysis

Of the 73 OA+SR participants assessed at all 3 time points, 53 (72.6%) were classified as actually using the OA program, as defined by 2 or more logins in the first 90 days of the study. In contrast, of the 58 SR only participants assessed at all 3 time points, 51 (87.9%) were classified as having been actually treated, as defined by 2 or more SR meetings attended. This difference in rates of actual use of the treatment options available approached significance, χ^2^_1_=3.75, *P*=.053.

We conducted separate repeated-measures analyses of variance on the 3 primary dependent variables to assess the effects of the between-subjects factor of treatment condition (ie, OA+SR or SR) and the within-subjects factor of time. We also had 2 a priori contrasts in the within-subject factor: the improvement from baseline to the average of the 2 follow-ups; and the change from the 3-month follow-up to the 6-month follow-up. As we found in our 3-month data, the improvement over time on PDA was highly significant, *F*_2,128_=78.26, *P*<.001. The tests of the preplanned contrasts indicated that, as hypothesized, the improvement from baseline to the average of the post assessments was highly significant, *F*_1,129_=154.85, *P*<.001, and the change from 3 months to 6 months was nonsignificant overall, *F*_1,129_=1.09, *P*=.30. However, in contrast to our finding with the 3-month data, the test of the treatment x time interaction is now significant, *F*_2,128_=3.16, *P*=.046. Tests of interaction contrasts indicated that the improvement from baseline to the average of the follow-up was comparable in the 2 conditions, *F*_1,129_=0.10, *P*=.92, but the change from 3 months to 6 months was significantly different in the 2 conditions, *F*_1,129_=6.32, *P*=.01. The reason for the latter finding, as seen in Table 2 below, is that while the SR only participants continued improving from 3 months to 6 months, the OA+SR group regressed slightly.

**Table 2:** Means (and standard deviations) and within-group effect sizes for each outcome variable for each treatment condition.

Variable and group		Baseline, mean (SD)	3-month follow-up, mean (SD)	6-month follow-up, mean (SD)	Improvement from baseline to average follow-up, mean	Improvement from 3 to 6 months, mean	Within group effect size *d*^a^
**Percent days abstinent**							
	OA^a^+SR^b^ (*n*=73)	42.13 (29.01)	74.03 (30.65)	67.28 (33.64)	28.53	−6.75	0.98
	SR only (n=58)	43.26 (29.11)	69.92 (32.43)	72.72 (31.57)	28.06	2.80	0.97
**Mean standard drinks per drinking day^d^**							
	OA+SR (n=73)	7.64 (4.45)	4.33 (3.70)	5.08 (5.20)	2.56	−0.75	0.65
	SR only (n=59)	8.19 (4.61)	4.82 (4.77)	3.99 (4.84)	4.20	0.83	0.84
**Inventory of Drug Use Consequences recent score^e^**							
	OA+SR (n=73)	39.37 (17.43)	19.01 (17.78)	19.88 (21.52)	19.92	−0.87	1.08
	SR only (n=58)	41.25 (19.72)	20.24 (19.50)	19.58 (21.27)	21.01	0.66	1.14

^a^Cohen *d* for change from baseline to average of 3-month and 6-month follow-ups *.*

^b^OA: Overcoming Addictions.

^b^SR: SMART Recovery.

^d^Standard drink is equal to 12 oz (355 mL) of 5% beer, 5 oz (149 mL) of 12% wine, or 1.5 oz (44 mL) of 80 proof liquor.

^e^Alcohol-related problems.

The DDD variable showed a similar pattern, although the treatment x time interaction did not reach significance. Thus, the improvement over time was highly significant, *F*_2,129_=36.88, *P*<.001, with again the significant improvement occurring from baseline to the average follow-ups, *F*_1,130_=72.95, *P*<.001, and the change from 3 months to 6 months being nonsignificant overall, *F*_1,130_=0.01, *P*=.93. The treatment x time interaction did not quite reach significance, *F*_2,129_ =2.53, *P*=.08. However, the pattern again was for the improvement from pre to the average of the posts to be comparable across conditions, *F*_1,130_=1.15, *P*=.29, but between 3 months and 6 months the SR only group continued to improve whereas the OA+SR group regressed slightly, though the test of the interaction contrast assessing differential change across groups did not reach significance, *F*_1,130_=3.37, *P*=.07.

The alcohol-related problems measure (InDuC) showed the same sharp improvement from pre to post, but in contrast to the other 2 dependent variables, there was no evidence of a treatment x time interaction. The improvement over time was highly significant, *F*_2,129_=59.96, *P*<.001, with again the significant improvement occurring from baseline to the average follow-ups, *F*_1,130_=120.86, *P*<.001, and the change from 3 months to 6 months being nonsignificant overall, *F*_1,130_=0.01, *P*=.95. The treatment x time interaction did not approach significance, *F*_2,129_=0.20, *P*=.82.

The mean within-group effect size was in the large range (ie, greater than 0.8), with a range 0.65-1.14. The largest effect sizes were in the domain of alcohol-related problems.

In addition to the primary analyses we ran on participants having complete follow-up data, we also analyzed data using maximum-likelihood mixed model methods to allow use of data from all participants, including those having missing data. Results were similar to those reported above. The omnibus test of the main effect of time was again significant for all 3 dependent variables, and the main effect of treatment was again nonsignificant for all 3 dependent variables. The omnibus test of the treatment x time interaction approached significance for PDA and DDD (.05≤ *P* ≤.10). Tests of contrasts agreed with the repeated-measures analyses in indicating that the time main effect was due to the improvement from baseline to the average of the postassessments on all dependent variables (*P*<.001) and that the evidence for a treatment x time interaction was due to the improvement from 3 months to 6 months being greater in the SR only condition than the OA+SR condition, both on PDA (*P*=.02) and on DDD (*P*=.06).

### Tests of Noninferiority

Although none of the tests of the null hypothesis of no difference between the OA+SR group and the SR only group in improvement from baseline to the average of the follow-ups approached significance for any of the dependent variables (*P* > .15), this failure to reject the null hypothesis is different from being able to confidently assert equivalence of the 2 treatments or noninferiority of the OA+SR treatment to the SR only treatment [56]. Thus, explicit tests for noninferiority were conducted where rejection of a null hypothesis that the OA+SR treatment was inferior to the SR only treatment would be a possible outcome [57]. Given a difference between treatments in amount of improvement between baseline and the post average corresponding to a small effect (*d*=0.2) might have been regarded as clinically significant, we set the margin of equivalence or noninferiority to one half of this amount or 0.1 of a pooled standard deviation. Using the pooled standard deviation on the original dependent variable, this *d* value of 0.1 was translated into a noninferiority margin for improvement from baseline to the average post score on each of our 3 dependent measures. Computing all differences so that the difference in improvement would be positive if the OA+SR group showed more improvement than the SR only group the mean difference in improvement for PDA was −3.32, 90% CI −11.78 to 5.13 whereas the noninferiority margin was −3.12; for mean drinks per drinking day, the mean difference in improvement was −1.29, 90% CI −2.80 to 0.23 with a noninferiority margin of −0.46; and for the alcohol-related problems measure, the mean difference in improvement was −3.48, 90% CI 10.54-3.59 with a noninferiority margin of −1.81. Noninferiority of OA+SR would have been demonstrated if the lower limit of the confidence interval had been greater than the noninferiority margin. However, in all 3 cases, not only the lower limit of the confidence interval but the mean difference itself was below the noninferiority margin. Thus, noninferiority is *not* established, meaning the result is inconclusive. Although tests of standard null hypotheses indicated we could not claim the predicted significant difference between the 2 conditions, we cannot confidently assert that the OA+SR treatment is not inferior to the SR only treatment.

### Actual-Use Analyses

We also conducted post hoc analyses based on participants’ actual use of the interventions: the between x within analysis of variance assessing the effects of the treatment condition, ie, OA+SR, OA only, or SR only, and time. As with the initial intent-to-treat analysis, separate analyses were conducted on each of the 3 primary dependent variables, with 2 a priori contrasts in the within-subject factor being of interest, namely, the improvement from baseline to the average of the 2 follow-ups, and the change from the 3-month follow-up to the 6-month follow-up.

There were 22 participants who reported only using the OA program, some despite having SR available to them subsequent to enrollment; in this sense they were self-selected for this group analysis. These 22 participants in the OA only group did not attend any SR meetings but completed 2 or more of the OA modules. This group was compared with a second group consisting of the 40 participants in the OA+SR condition who completed 2 or more OA modules and who also attended 2 or more SR meetings, as well as with a third group consisting of the 61 participants from the OA+SR condition and the SR only condition who did not complete any OA modules but who attended 2 or more SR meetings. These 3 groups, OA only, OA+SR, and SR only did not differ significantly by gender, ethnicity, age, or education. Although there were no significant differences between these groups in mean baseline values on our 3 primary dependent variables, the trend in each case was for those in the OA+SR group to be less impaired initially than those in the OA only group.

**Figure 2:**
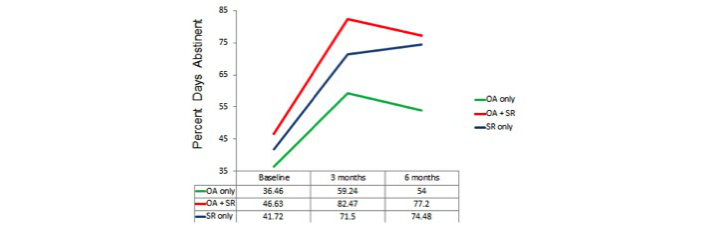
Actual use analysis: percent days abstinent. OA: Overcoming Addictions; SR: SMART Recovery.

**Figure 3:**
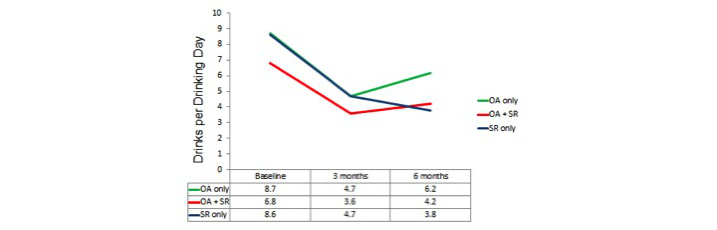
Actual use analysis: drinks per drinking day. OA: Overcoming Addictions; SR: SMART Recovery.

**Figure 4:**
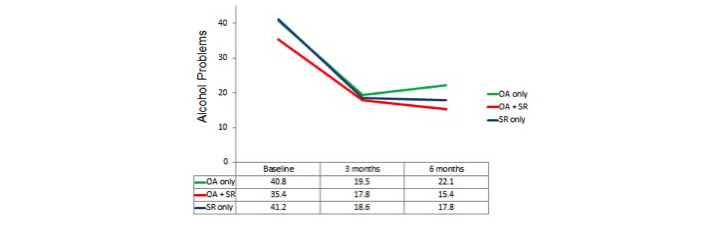
Actual use analysis: alcohol-related problems. OA: Overcoming Addictions; SR: SMART Recovery.

Repeated-measures analyses of variances again indicated highly significant changes over time on all 3 dependent variables (*P*<.001) with the locus of the effect being the improvement from the baseline to the average of the post measures (*P*<.001) but there being no significant overall change from 3 months to 6 months. Results for these 3 groups defined by actual use, that is, OA only, OA+SR, and SR only, are shown in Figures 2,3, and 4.

The tests of the group x time interaction were not significant, although there was a trend for an interaction on PDA, *F*_4,208_=2.06, *P*=.09 (Figure 1). Tests of interaction contrasts indicated that the locus of evidence for an interaction was that the change from 3 months to 6 months in the SR only group was significantly different from that in the OA+SR and OA only groups, *F*_1,105_=4.31, *P*=.04.

Similarly, for DDD, although the omnibus test of the group x time interaction was nonsignificant, *F*_4,210_=1.76, *P*=.14 (Figure 2). The test of the same interaction contrast suggested a trend for the continued improvement in the SR only group to be different from the decline in the OA groups between 3 and 6 months, *F*_1,40_=3.18, *P*=.08.

### SMART Recovery Meetings or Other Support

Participation by those in the SR only condition in SR meetings, both face-to-face and online, declined sharply between the first 3 months and second 3 months of the follow-up period. Participants in the SR only condition reported attending 3.17 face-to-face meetings in the 3 months after baseline but only 1.86 in the next 3 months, *t*_58_=3.35, *P*<.001; similarly, online meetings attended declined from 5.85 to 3.02, *t*_58_=4.00, *P*<.001. Because 78% and 66% did not attend, respectively, any face-to-face or online meetings between the 3- and 6-month follow-ups, the frequency of meetings attended in the first 3 months was used to assess evidence for dose-response relationships. Although as we previously reported [23], number of face-to-face meetings attended in the 90 days after baseline had been significantly positively related to improvement from baseline to 3 months on all 3 of our outcome variables, it was found now to be negatively related to improvement from 3 to 6 months on PDA (*r*=−.082), DDD (*r*=−.246), and InDUC (*r*=−.050). The number of days of counselor visits, other meetings, or any support also was negatively nonsignificantly related to improvement from 3 to 6 months. Number of online meetings, in contrast, was at least positively, though nonsignificantly, related to improvement from 3 to 6 months on our dependent measures (0.050, PDA; 0.112, DDD; 0.083, InDUC).

### Number of Overcoming Addictions Sessions

In the OA conditions, participation in OA, as measured by number of logins to the website, declined from 7.31 on average in the first 3 months to 1.29 in the next 3 months, *t*_72_=10.19, *P*<.001. Attendance in the SR meetings by OA participants also declined, though the change in participation was not significantly different from that seen in the SR only condition. Interestingly, whereas number of OA logins had been only weakly and nonsignificantly related to improvement from baseline to 3 months on our dependent variables, the use of the OA site during the first 3 months was more strongly predictive of improvement from 3 to 6 months. Specifically, OA logins in the first 90 days after baseline correlated .359 (*P*=.005) with improvement in PDA from 3 to 6 months and .352 (*P*=.006) with improvement in InDUC. We also examined the number of OA modules actually completed by participants(mean 6.39, SD 4.28). The OA modules completed also was predictive of improvement from 3 to 6 months on PDA (*r*=.297, *P*=.02) and InDUC (*r*=.332, *P*=.007). In addition, the number of modules completed was associated with final levels on all three dependent variables: PDA, *r*=.263, *P*=.04; DDD, *r*=−.292, *P*=.018; and InDUC, *r*=−.362, *P*=.003.

### Corroboration of Self-Reported Drinking by Significant Others

Data were available at all 3 time points from 97 significant others (SOs) on 2 of our primary dependent variables, PDA and DDD. Examining the effects of time and treatment on these SO reports generally corroborated the clients’ self-reports in that the tests of change over time were highly significant, for PDA, *F*_2,94_=63.49, *P*<.001, and for DDD, *F*_2,94_=65.59, *P*<.001, and the test of the treatment x time interaction were nonsignificant, *P*>.2. Although the SOs’ reports were similar to those of the clients in perceiving by far the greatest change was from baseline to the average of the follow-ups, *F*>100, *P<*.001, the reports differed in that the SOs thought there was continued improvement from 3 months to 6 months in both groups whereas the clients reported improvement only in the SR only condition. For example, SOs reported clients continued to improve significantly from 3 months to 6 months in PDA, *F*_1,95_=6.84, *P*=.01, and reported PDA increased from 76.4 to 84.6, whereas these corresponding participants reported their PDA declining nonsignificantly from 70.3 to 70.1. The correlations between SO and client reports which ranged from .57 to .69 at baseline and 3 months declined at 6 months to .46 for PDA and to .31 for DDD.

### Individual Differences Among Participants: Readiness to Change

In addition to examining treatment effects, we conducted additional analyses of participants’ behavioral changes already under way at the time of entry into these programs. A majority (127/188, 67.6%) of the participants enrolling in the study had gone more than one day without drinking immediately before enrolling in the study. The number of days since the last drink in this subgroup ranged from 2 to 84 days before enrolling, with a mean of 15.6 and a median of 10.0. The number of drinks on that last day of drinking before enrollment was much *greater* for those who had been abstinent for more than a day (mean 9.6, SD 6.2) than for those who had been drinking on the day before enrollment (mean 6.0, SD 4.0), *t*_171.5_=4.76, *P*<.001. Similarly, those who had been abstinent for more than a day reported a higher level of mean drinks per drinking day over the previous 3 months, 8.65 versus 6.74, *t*_186_=2.87, *P*=.005. However, those who had taken a break from drinking alcohol reported a much higher PDA for the 90 days before intake than the nonabstinent group, 53.1% versus 25.7%, *t*_186_=6.75, *P*<.001. The 2 groups did not differ in terms of recent alcohol-related problems on InDUC, 41.9 versus 40.1, *t*<1.

**Figure 5:**
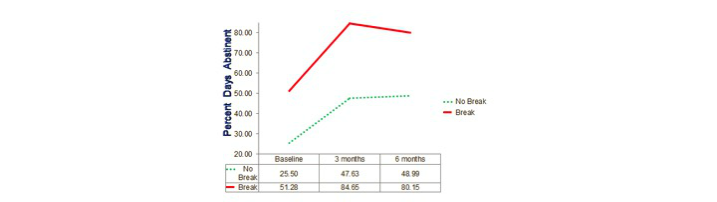
Stage of change analysis: percent days abstinent.

**Figure 6:**
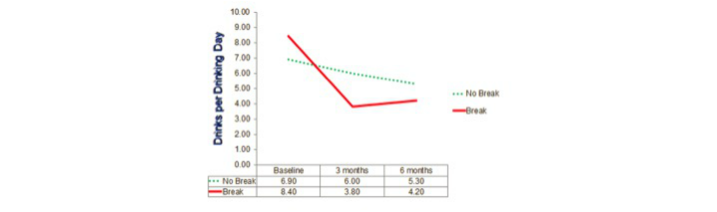
Stage of change analysis: drinks per drinking day.

The importance of differences among participants was assessed in intent-to-treat repeated-measures analyses of variance with 2 between-subjects factors of treatment condition (OA+SR vs SR only) and whether the participant had had his or her last drink more than 1 day before enrollment (break or no break), and the one within-subject factor of time, using number of drinks on the last day of drinking, centered at its grand mean as a covariate. Means are plotted for the 3 outcome variables in Figures 4,5, and 6. For PDA, the break factor did not interact with treatment or time; however, the main effect of this between-subject factor of break was very highly significant, *F*_1,126_=50.7, *P*<.001. There was a trend for the advantage of the break group over those still drinking to increase from 25.7 PDA at baseline to 34.1 PDA at the average of the postassessments, but this difference did not reach significance, *F*_1,126_=2.77, *P*=.099

With DDD, in addition to the strong between-subject main effect of break, *F*_1,127_=8.79, *P*=.004, 2 other effects were significant. The interaction of break with time was clearly significant for DDD, *F*_2,126_=4.57, *P*=.01, as was the interaction of time with the covariate of number of drinks on last day of drinking, *F*_2,126_=20.21, *P*<.001. As suggested by Figure 6, the reason for the break x time interaction was that although there was little difference at baseline in DDD across groups, the group that had been abstinent for more than one day before enrollment decreased their mean drinking levels much more than those who were drinking on the day before enrollment. For those who taken a break for more than a day, DDD declined from 8.41 at baseline to 4.02 averaging across the 2 postassessments, whereas for the other group the decline was only from 6.87 to 5.63, *F*_1,127_=9.08, *P*=.003 (Figure 6).

The reason for the significant time x number of drinks on last day of drinking was that participants who were drinking more just before enrollment decreased their DDD significantly more from baseline to the average of the postassessments, *r*=.485, *P*<.001. However, those who were drinking *less* on their last day of drinking improved more from 3 months to 6 months, as the number of drinks on last day of drinking correlated significantly negatively with the improvement (ie, decrease) in DDD from 3 months to 6 months, *r*=−.274, *P*=.002.

**Figure 7:**
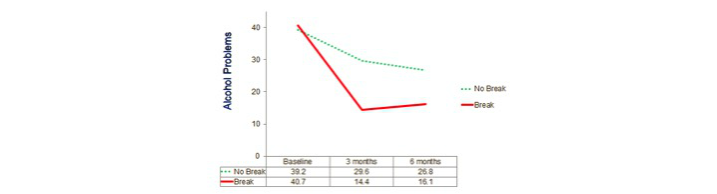
Stage of change analysis: alcohol-related problems.

The pattern on InDUC Recent Total was essentially the same as that for DDD. That is, not only was the between-subject main effect of break highly significant, *F*_1,127_=14.81, *P*<.001, but the interaction of break with time was again significant for InDUC, *F*_2,126_=6.19, *P*=.003, as was the interaction of time with the covariate of number of drinks on last day of drinking, *F*_2,126_ (2,126)=4.06, *P*=.02. Again, as shown in the figure below, those who had taken a break improved more from baseline to the postassessments on InDUC, with the break group declining from 40.7 to 15.2, and those drinking the day before enrollment only declining from 39.2 to 28.2. As before, the reason for the time x covariate interaction was that number of drinks on the last day of drinking correlated significantly positively with improvement from baseline to the average of the postassessments, *r*=.215, *P*=.01, but significantly negatively with improvement from 3 months to 6 months, *r*=−.212, *P*=.02 (Figure 7).

As might be concluded from the plots above, mean within-group effect sizes differed greatly across these 2 subgroups of participants (see Table 3). Whereas the mean *d* across the dependent variables was 0.51 (a medium effect size) for participants who had been drinking on the day before enrollment, for those who had not been drinking just before enrollment the mean *d* was more than twice as large (1.24, or more than 50% greater than Cohen’s cutoff for a large effect).

**Table 3:** Means (and standard deviations) and within-group effect sizes for each outcome variable for subgroups of participants that had or had not stopped drinking for more than one day before enrollment.

Variable and group		Baseline, mean (SD)	3-month follow-up, mean (SD)	6-month follow-up, mean (SD)	Improvement from baseline to average follow-up, mean	Within group effect size *d*^a^
**Percent days abstinent**						
	Break (n=87)	51.28 (26.57)	84.65 (21.84)	80.15 (25.39)	31.12	1.26
	No break (n=44)	25.53 (25.90)	47.63 (33.09)	48.99 (35.90)	22.78	0.71
**Standard drinks per drinking day^b^**						
	Break (n=87)	8.41 (4.81)	3.80 (4.21)	4.25 (5.32)	4.39	0.91
	No break (n=45)	6.87 (3.73)	5.99 (3.84)	5.26 (4.47)	1.25	0.31
**Inventory of Drug Use Consequences recent score^c^**						
	Break (n=87)	40.71 (18.73)	14.37 (14.37)	16.07 (18.06)	25.49	1.49
	No break (n=45)	39.24 (18.02)	29.60 (21.45)	26.84 (25.27)	11.02	0.51

^a^Cohen *d* for change from baseline to average of 3-month and 6-month follow-ups *.*

^b^Standard drink is equal to 12 oz (355 mL) of 5% beer, 5 oz (149 mL) of 12% wine, or 1.5 oz (44 mL) of 80 proof liquor.

^c^Alcohol-related problems.

### Participant and Intervention-Related Variables

In addition to analyzing outcomes according to treatment group and readiness to change, we conducted additional analyses on participants with access to OA in order to explore for the possible influence of factors germane to Web-based interventions. For each variable, separate repeated-measure analyses of variance were conducted on the 3 primary dependent variables (ie, InDuC, PDA, and DDD).

We asked participants to estimate how much time per week they spent on the Internet—at work or school, at home, and elsewhere (café, library, etc). We totaled these estimated hours to create a continuous variable characterizing the participant’s relative fluency with the Internet. The continuous moderating variable of average amount of time on the Internet per week was zero-centered. Participants with access to OA reported spending an average of 23.0 (SD 16.8) hours per week on the Internet. Analysis indicated that the impact of participant’s fluency with the Internet did not significantly impact treatment outcomes: PDA, *F*_2,63_=1.004, *P*=.37; DDD, *F*_2,63_=0.983, *P*=.38; or the InDuC, *F*_2,63_=0.029, *P*=.97.

To test for the impact of the user’s sense of how easy the site was to navigate and use, we examined their responses to the pertinent questions in the exit interview for shared variance and created a new variable —“ease factor”— (that was also z-centered) to test for the impact of this factor. Analysis indicated that participant’s subjective rating of how easy it was to navigate the OA site did not significantly impact treatment outcomes: PDA, *F*_2,44_=0.55, *P*=.58; DDD, *F*_2,44_=1.21, *P*=.31; or the InDuC, *F*_2,63_=1.029, *P*=.34. To see whether user’s satisfaction with the amount of content on the site had an effect on outcomes, we collapsed the 3 possible responses on the exit interview (ie, too much, too little, just right) into a single, dichotomous variable indicating either a satisfactory or unsatisfactory amount of information. Analysis indicated that participant’s satisfaction with the amount of information on the OA site did not significantly impact treatment outcomes: PDA, *F*_2,44_=0.699, *P*=.50; DDD, *F*_2,44_=1.06, *P*=.34; or the InDuC, *F*_2,63_=0.010, *P*=.99.

Finally, we asked all participants in the study to report which treatments, influences, and/or other factors they had used or encountered throughout the duration of the clinical trial with respect to changing their drinking behavior (Tables 4 and 5). Participants were allowed to make as many attributions as they wanted to. Results showed that a majority of the study participants interviewed indicated that both SR and OA were influential in helping them to make changes to their drinking. It is also clear that study participants made use of a variety of therapeutic resources in addition to OA and SR.

**Table 4:** Participants’ attribution to factors that helped them make changes to their drinking.

Treatment or influence	OA^a^ SR^b^	Most helpful	Very helpful	Some help	No help	Total positive
SMART face-to-face	OA	7	12	3	43	22
	SR	8	5	2	41	15
SMART online	OA	2	16	8	41	26
	SR	13	17	3	42	33
Overcoming Addictions	OA	14	19	15	20	48
					
Alcoholics Anonymous	OA	3	5	1	6	9
	SR	1	7	0	1	8
Other treatment program	OA	3	2	2	0	7
	SR	2	3	0	1	5
Personal therapist	OA	4	7	2	1	13
	SR	6	7	3	0	16
Self-determination	OA	20	4	1	0	25
	SR	11	8	0	1	19
Some other factor	OA SR	12 12	12 8	8 3	1 1	32 23

^a^OA: Overcoming Addictions.

^b^SR: SMART Recovery.

**Table 5:** Other factors cited as helpful.

Treatment or influence	OA^a^ SR^b^	Number citing
Social support	OA	11
	SR	7
Changed thinking or awareness	OA	9
	SR	5
Joining the randomized controlled trial	OA	7
	SR	3
Medication	OA	3
	SR	5
Just did it	OA	2
	SR	3

^a^OA: Overcoming Addictions.

^b^SR: SMART Recovery.

## Discussion

### Principle Findings

We compared 2 treatment modalities based on the cognitive behavioral intervention in SR. One modality (SR) is social in nature (ie, entailing meetings either in person or online), while the other (OA) is self-directed. We hypothesized that the structured and personalized design of OA would lead to superior outcomes to SR and that it would enhance outcomes even further when coupled with SR. This was not the case. The experimental hypotheses were: (1) Both groups will reduce their drinking and alcohol or drug-related consequences at follow-up compared with their baseline levels; and (2) The OA+SR condition will reduce their drinking and alcohol or drug-related consequences more than SR only. These results support our first experimental hypothesis but not the second.

On average, all participants improved on outcomes that are important to recovery from problem drinking. They significantly increased the percentage of days they were abstinent over the 6-month follow-up period, significantly reduced the number of drinks they consumed on the days when they did drink, and experienced a marked reduction in alcohol-related problems. The mean effect sizes for reductions in drinking and alcohol-related problems, averaging across the 3 dependent variables, were in the large range (0.8+). These statistically significant results are also clinically significant. We consider it remarkable that participants with this degree of heavy drinking, and reporting a significant level of clinical distress, made and largely maintained these changes over the follow-up period of 6 months. Our decision not to include a no-treatment control in this trial precludes us from making direct causal attributions about the effectiveness of OA. Nonetheless, the results of this trial provide support for the use of both OA and SR, and more generally, the hypothesis that Web-based interventions based on evidence-based treatments can be helpful even for heavy drinkers.

It is clear that participants used the intervention modalities and components available to them according to their own inclinations. Some participants preferred using the Web application alone, some preferred to attend meetings, and many chose to utilize both. Unlike patients provided with “traditional” guided and structured psychotherapy, participants used intervention components as much as they felt they needed to, when they needed to, and reported acquiring the skills and techniques of the SMART model in both the social and self-directed modalities. The heterogeneity in how participants used and benefitted from the intervention presents a stark contrast to the rigidity of structure typically prescribed in evidence-based therapies. Moreover, perhaps the more striking discovery we made during our exit interviews was that participants’ engagement with their recovery was reflected not just in their use of SR or OA alone, but also in the plethora of other means they utilized to support changes in their drinking, concomitant with their use of SR and OA. We feel there are important implications in this finding that bear on the development, implementation, and testing of Web-based interventions.

Similarly, the fact that individuals enrolling in the study were often unwilling to accept assignment to the OA only condition also indicates that they already had a sense of what they wanted to facilitate their recovery. The participants in the study were obviously attracted to SR because of its theoretical approach and the tools it offers, but many of them also wanted a social milieu of some sort through which they could acquire or process these intervention components, regardless of the benefits available through a Web-based intervention. Further, participants who used SR, either alone or in conjunction with OA, exhibited a trend toward slightly better outcomes at the 6-month follow-up than those who used OA exclusively, although this finding was not statistically significant. Given this, our results are consonant with other studies finding that, for some people at least, stand-alone Web-based interventions are more effective when combined with some form of social support or learning. Apart from their advantages, there may be limits to both the appeal and the effectiveness of self-guided Web-based interventions for problematic alcohol use—even those based on EBTs.

While it is good to know that participants in the trial were able to use and benefit from the interventions made available to them in the study, the exit interview results suggest that the conditions required to establish an evidence base for free-standing computer-delivered interventions are inherently equivocal. The remote context of their use and testing make it very difficult, if not impossible, to design an ecologically valid study that could control for sort of treatment foraging exhibited by the participants in this study—even if a no-treatment control group were included in the design. While others have made this observation before [10], in this study, we found strong qualitative data to support this conjecture. It may be the case that not only do people engage in Web-based treatments differently than they do in conventional treatments, but they might also be simultaneously seeking and using other therapeutic resources differently as well. While this state of affairs does present methodological challenges for researchers and intervention developers, it does not obviate the benefits of these treatments. The results of this study support the theory that having different ways to learn about and use the evidence-based tools in the SMART Recovery protocol gives problem drinkers clinically sound options with regard to how they learn to achieve and maintain abstinence. Although researchers typically do not design interventions with evidence-based components to be self-directed, our findings support a therapeutic picture in which having online resources available increase the chances that individuals can find a path to recovery that suits them.

Regardless of which intervention was utilized by study participants, evidence for the added benefit of increased engagement with either OA or SR was limited—as it often is when testing Web-based interventions. The results here contradict the conventional perspective that more treatments, and more structured treatments, facilitate better outcomes. The one notable exception we found to this trend was the “sleeper effect” we detected in the second half of the trial among individuals who made greater use of OA in the first 3 months. While we consider this a positive finding for OA, we can only speculate as to why this was the case. It may be that the cognitive-behavioral tools offered in OA require time and practice to produce gradual but lasting change. It may also be the case that participants in the OA group exhibited assessment reactivity to the 3-month follow-up. The 3-month interview was the first time when participants in the trial were asked to quantify and characterize their drinking, and for individuals who were provided access to OA this may have been the first time they addressed their problem drinking in a social context. The session could have motivated them to renew their attempts to change their drinking behavior. If so, this would corroborate the basis for ongoing questions about the relationship of both social interactions, as well as assessment and feedback protocols, to the effectiveness of Web-based interventions for problematic alcohol use [2,3,14].

### Influence of Participant and Intervention Factors on Outcomes

Our findings indicate that the individuals who had taken steps to stop drinking before joining the study were primarily responsible for the changes over time that we found in both the OA+SR and SR only conditions. SR and OA provided resources for individuals in the action and maintenance stages of change, and those resources both encouraged the nascent steps and supported their durability. A basic tenet of Prochaska and DiClemete’s model is that individuals in the action and maintenance stages of change are motivated to use clinical tools (eg, functional analysis, problem-solving exercises) that have been shown to be effective in helping people to confront the challenges they face. Both SR and OA make these resources available and participants in the study who were in the action stage of change made use of them. Based on the evidence, it is also fair to assert that individuals who came into the study without yet having quit drinking were not helped as much with their desire to do so by either OA or SR. The fact that Web-based interventions are associated with positive outcomes among drinkers who are actively seeking resources to support their behavior change, but less so for individuals who are not yet at that stage, should inform their deployment in stepped-care programs, and thus better substantiate their implementation as part of an overall public health strategy.

We found no evidence for the impact of other participant or intervention-related variables thought to influence the effectiveness of Web-based interventions. None of the identified factors related to Web-based interventions (fluency with the Internet, participants’ subjective ratings of how easy the site was to navigate, nor satisfaction with the amount of content on the site) exhibited any influence on treatment outcomes. We believe that there are 2 reasons that may account for this negative finding. The first derives from the relatively high level of education reported by participants in this study. Researchers have found that more highly educated individuals are slightly more likely to benefit from Web-based interventions [1,28], in part because more educated individuals tend to solve problem more persistently and effectively when confronted with navigational challenges on websites [40]. Additionally, findings of the Pew Research Center’s Internet and American Life Project show that older adults have accessed the Internet at increasing rates over the last 15 years [58]. It is likely the case that as time has passed, the skills required to navigate the Internet have become ever more commonplace, and the structure of websites, whether confused or intuitive, guided or self-directed, have become less and less of a mitigating factor to individuals who seek to access their content.

Finally, one other aspect of this study that we feel deserves mention, and which is consistent with our prior studies of Web-based interventions [24], is the high participation rate (61%) of women. This result is consonant with the ability of Web-based interventions to reach historically underserved populations. Women have greater perceived barriers to treatment than men do. Brady and Ashley [55] reported that women are more likely to report economic barriers and family responsibilities when seeking treatment. Many women realizing the need for treatment are more likely turn to Web applications for help before they seek individual treatment due to gender differences in stigmatization for treatment of alcohol use disorders with shame, embarrassment, and discouragement from family members being more commonly reported by women than by men [59,60]. In addition, women experience the salience of multiple roles (eg, career, mother, spouse, friend) and find they must prioritize their time in the most efficient way possible. OA and online SR meetings are typical of interventions that can provide alternative interventions for alcohol problems that do not impact other roles in the same way as seeking individual treatment.

Analysis of Internet usage and average time spent on the Internet supports our conclusions for the differences in demographics. Slightly more women in North America use the Internet than men and for greater amounts of time [61]. Although Internet usage differs according to the category of activity or personal interest for women and men, health sites are visited more frequently by women (22.8% and 17.4%, respectively). In addition, women across a wide age range (eg, age 15-55+ years) are more apt to turn to community resources available on the Internet at greater rates than men. Even though OA and SR are not tailored to differentially attract women or men, the prevalence of women in our study is consistent with the literature on women turning to the Internet for health and community resources.

### Limitations

There are a number of limitations to this study. First, as noted, we did not include a no-intervention control group in our study design. While we found it neither ethically appropriate nor practically feasible to include such a group, the lack of a comparison prevents us from being able to control for individuals whose prior decision to stop drinking was largely responsible for the improvement observed in this study. In addition, we could not separate out the effects of assessment reactivity that, based on participants’ anecdotal reports, did sometimes occur as a function of the follow-up evaluation. Third, the relatively small sample size as well as the high level of education (mean 16 years) reported by participants in the study potentially limit the generalizability of the outcomes in populations with lower levels of education. Fourth, the requirement for an SO to corroborate the participant’s self-report of drinking may have further limited the generalizability of this sample’s results. We considered that requirement necessary though, as we had no other way to confirm participants’ self-reports of their drinking. Another limitation is that we were not able to randomize a full complement of participants into the OA only group, which meant that this group was essentially self-selected and that the small sample size of the OA group limited the power of the analyses.

When analyzing variables thought to moderate outcomes (eg, participant characteristics), the ideal method would be to assess them directly before beginning the processes they are thought to influence. Our posthoc assessment, while acceptable given the lack of feasible alternatives, was likely mediated by the participants’ recollection of the site. One can’t help but wonder whether a follow-up coming closer on the heels of the participant’s disengagement from the site might not have yielded more vivid recollections of what it was like for individuals to use it. Further, given the exploratory nature of this study, it was unclear whether the constructs we intended to tap were in fact done so with as great a precision as might be hoped. There may be less intrusive, more ecologically valid methods to probe how participants engaged the site, and how this engagement mediated outcomes on the variables of interest. Indeed, this question lies at the vanishing point where the development of, and research into, the next generation of these interventions converge.

### Future Research

The results of this study did influence the subsequent development of OA. Even though navigation was not found to impact outcomes, anecdotal feedback from participants as well as their lower than expected uptake of the intervention motivated us to revise the site. We developed an automated program that sends users an email each week, prompting them to log into the site. The email briefly describes the week’s “lesson,” and an embedded link takes the users directly to that page in the site, after first allowing them to enter their urge data for the week. We also added linked summary statements in the headers of each module and provided more “tunneling” within the site in response to the feedback of participants who desired more guidance through the site. Finally, we have created new interventions that combine OA with *The Drinker’s Check-up* [62,63] hypothesizing that the hybrid design will increase users’ motivation for engaging the self-directed exercises in the OA program.

Having said that, our null results with regard to the possible moderating and mediating effects of interface with a Web-based intervention suggest that although low Internet fluency may have at one time presented a significant barrier to accessing these treatments, it may be less of an issue as the revolution in media technology proceeds, at least among more highly educated individuals. Similarly, while it is important that research into website and participant factors continues, and that researchers continue to develop innovative methodologies to test each new generation of interventions [64], we believe that improving their effectiveness will also benefit from an investigation into the novel ways that individuals approach Web-based interventions differently than they do “traditional” treatments. Broader exploration of all the recovery behaviors of individuals who use Web-based treatments for problem drinking may inform current assumptions about both the development and implementation of these interventions, and thus help to solve the riddle that currently links user engagement with clinical outcomes.

### Summary

Web-based interventions for heavy drinkers are not as unfamiliar as they once were, but a decade of intensive research and development has left many unanswered questions about their effectiveness. The adaptation of evidence-based techniques and treatments to this relatively new mode of delivery is complicated by the pace and dispersion of technological innovation, human-user adaptation to those innovations, and a lack of evidence to clearly guide the appropriate deployment of Web-based behavioral resources.

This study found evidence of a positive treatment effect for the OA site. The evidence did not detect an added benefit of OA over the preexisting SR intervention upon which it is based, in that it neither surpassed nor enhanced its effectiveness. There was evidence to show that OA can serve as a feasible alternative to SR, and as a Web-based intervention, it entails the advantages of access, reach, and cost-effectiveness. Further, our results suggest that Web-based interventions work particularly well for individuals who are actively making changes to their drinking behavior.

## Abbreviations

AUDIT: Alcohol Use Disorders Identification Test
BSI: Brief Symptom Inventory
DDD: mean standard drinks per drinking day
DWI: driver while intoxicated
InDUC: Inventory of Drug Use Consequences
OA: Overcoming Addictions
PDA: percent days abstinent (in previous 90 days)
SO: significant other
SR: SMART Recovery
